# Development of an Expert System as a Diagnostic Support of Cervical Cancer in Atypical Glandular Cells, Based on Fuzzy Logics and Image Interpretation

**DOI:** 10.1155/2013/796387

**Published:** 2013-04-18

**Authors:** Karem R. Domínguez Hernández, Alberto A. Aguilar Lasserre, Rubén Posada Gómez, José A. Palet Guzmán, Blanca E. González Sánchez

**Affiliations:** ^1^Instituto Tecnológico de Orizaba, Avenida Oriente 9 No. 852, Colonia Emiliano Zapata, 94320 Orizaba, VER, Mexico; ^2^Hospital Regional de Río Blanco, Entronque Autopista Orizaba-Puebla km 2, 94735 Río Blanco, VER, Mexico

## Abstract

Cervical cancer is the second largest cause of death among women worldwide. Nowadays, this disease is preventable and curable at low cost and low risk when an accurate diagnosis is done in due time, since it is the neoplasm with the highest prevention potential. This work describes the development of an expert system able to provide a diagnosis to cervical neoplasia (CN) precursor injuries through the integration of fuzzy logics and image interpretation techniques. The key contribution of this research focuses on atypical cases, specifically on atypical glandular cells (AGC). The expert system consists of 3 phases: (1) risk diagnosis which consists of the interpretation of a patient's clinical background and the risks for contracting CN according to specialists; (2) cytology images detection which consists of image interpretation (IM) and the Bethesda system for cytology interpretation, and (3) determination of cancer precursor injuries which consists of in retrieving the information from the prior phases and integrating the expert system by means of a fuzzy logics (FL) model. During the validation stage of the system, 21 already diagnosed cases were tested with a positive correlation in which 100% effectiveness was obtained. The main contribution of this work relies on the reduction of false positives and false negatives by providing a more accurate diagnosis for CN.

## 1. Introduction

During the last fifty years, the incidence and mortality rate for cervical cancer has decreased, mostly in developed countries thanks to the implementation of prevention programs (cytology). However, among gynecologic types of cancer, this pathology ranks second place in developing countries, mainly, in poorer zones. 

On the other hand, the risk factors linked to this disease are of the socioeconomic type, primarily consisting of multiple incidences in rural zones, as well as the young age of the patients. The human papillomavirus is considered to be a causal agent and is linked to the early beginning of sexual relations, especially with casual sex partners. 

In 2008, more than 80,000 women were diagnosed with cervix cancer and 36,000 died because of this disease in Latin America only. In developing countries, such as Mexico, CN is linked to an even higher mortality rate, contrary to rates in more developed countries. However, in Mexico, several studies have been performed in this field. Every year, the Mexican Institute of Social Security (IMSS in Spanish) diagnoses around 15,000 cases of CN [1].

This current research is based on specialists' expertise in diagnosing cervix cancer. The expert system models the information provided by doctors for the decision-making process in the diagnosis. Then, in a second phase, the system performs an analysis by segmenting the processed images to show the interest parameters in the cytological image suggested by the Bethesda system [2] for its diagnosis. In the first two phases, the system is integrated by a fuzzy logics model and its graphic interface in which a cytological image is uploaded and the interest data about the clinical records of a patient is typed out. Finally, a diagnosis is suggested for precursor injuries of CN. In cases dealing with doctor's expertise, an expert system that is able to diagnose positive or negative cases does not rise any interest; for this reason, the contribution of this work focuses on the atypical, where the information is not enough to emit either a positive or a negative CN diagnosis. These kinds of cases are known as atypical glandular cells (AGC). The expert system described herein is able to offer a diagnosis since it has mathematical information from the image processing, which a doctor cannot obtain when it is analyzed manually. With this information and the addition of the clinical records of a patient gathered in an expert system, it can be used as a support tool in the decision-making process for the specialists. As a result, a diagnosis for precursor injuries for atypical cases is obtained.

This work is structured in three parts. The first stage describes the doctor's perspective, where the studied illness is presented, as well as its factors and the diagnosis' methods. Furthermore, in the same stage, a review of the related literature in this topic is presented along with its main contributions. The second stage presents the development of this research among the three stages that comprise the expert system. Finally, in the third stage, a description of the case study is presented, and the tests are run on the already diagnosed cases in order to validate the system. 

## 2. Medic Perspective and a Literature Review

After breast cancer, worldwide, cervical cancer is the second most frequent cancer among women. Until 2008, an estimated of 529,800 deaths were related to this disease around the world [3]. Nowadays, cytological screening by means of Pap smear has reduced the cancer incidences in terminal stages. As a result, an important increase in precursor cancer injuries in asymptomatic women has been recorded. In their natural evolution, these injuries can be treated and eradicated if an appropriate diagnosis is available on time. 

Everyday, medical breakthroughs provide valuable information that boosts treatments and cures to a countless number of illnesses. However, the need for creation of multidisciplinary groups, which can provide different knowledge and perspectives about the same topic, is also evident. 

### 2.1. Cancer Manifestation

Cancer is a pathologic tissue growth originated due to a continual proliferation of abnormal cells. It can stem from any type of cell from different tissues in the human organism. 

There are many types of cancer. One of the most threatening to women is cervical cancer (CN), which unlike the others, if it is detected during its early stages, the possibilities for a total recovery are plenty [4]. More than 80% of deaths caused by CN, come from countries with high or medium levels of poverty. It is to be expected that the annual figure of deaths caused by CN will raise and exceed to 11 million by 2030 [5]. 

Cervical cancer's underlying cause is the infection by the human papillomavirus (HPV), which is a common sexually transmitted disease (STD). However, 10 or 20 years are needed for a precursor injury produced by HPV to be turned into invasive cancer. Unfortunately, it is estimated that 95% of women (in the reproductive age) who inhabit developing countries have never taken a Pap smear [6]. 

The total recovery rate for this disease is closely associated to the stage of development at the moment of the diagnosis and to the availability of its treatment, since CN is deadly if it remains untreated. Due to its complexity, CN treatment directly relies on its appropriate and accurate diagnosis. 

There is no question about the decisive role a doctor's expertise plays in order to emit a positive diagnosis. However, when dealing with cytology samples in which cellular presence is inconclusive to discard either a positive or a negative call, an exhaustive analysis is required. Although it is uncommon, atypical glandular cells come up as a big challenge to specialists since most of these samples cause a bigger contribution to call false positives or false negatives. It will always be worse to call a false negative in patients rather than a false positive, for obvious reasons. 

As discussed earlier, the contribution of this research focuses on the diagnosis of atypical cases of CN, through the study of atypical glandular cells (AGC). These types of cells present an atypical nucleus that exceeds the reactive or reparative changes but lacks endocervical adenocarcinoma in situ features. This expert system is expected to be a supporting tool in the decision-making process for cervical cancer diagnosis; the system is specially oriented to patients whose samples show AGC and as a consequence reduce false positives and false negatives within the diagnosis.

**Figure 1:**
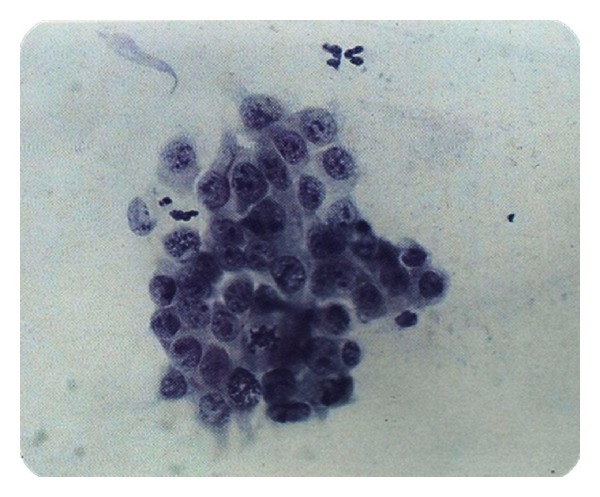
AGC.

In Figure 1, cytology of atypical endocervical cells is shown, where the nuclei almost triple their normal size, and a suggestive feature for adenocarcinoma can be observed. Despite this, nucleus pigmentation and nucleolus presence (small spots inside nuclei) are only observed in normal or nonmaligns samples.

**Figure 2:**
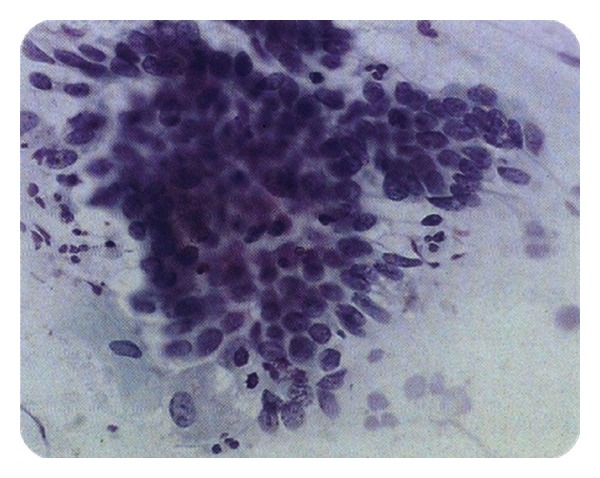
AGC, suggestive neoplastic.

In Figure 2, a suggestive neoplasic sample is presented, which means it's likely to be a positive Cancer diagnosis due to its mostly dark coloring and cellular fragmentation. However, the grouping and size of nucleolus suggest a normal sample.

This research was carried out with the collaboration of the Cytopathology Department of Rio Blanco's Hospital, where the decision-making process for a patient's diagnose is made under Mexico's General Hospital and NOM 014-SSA2 1994 procedures. These procedures consist of inspections of the sample by 2 different cytotechnologists that emit a diagnosis. If the cytotechnologists have different diagnosis, they discuss their point of views in order to reach an agreement and emit a diagnosis by consensus. In the first inspection (quick inspection done in less than one minute), all the present fields on the lamella are observed by a cytotechnologist, and the first diagnosis is emitted. Afterwards, in a slow inspection (at least four minutes), special emphasis is placed on the lamella fields that are not clear for injury rates. From this second inspection, a second diagnosis is also obtained. If a positive correlation exists between both diagnoses, a final diagnosis is emitted. Otherwise the sample goes through a consensus in order to get a final result. In the first two inspections, two cytotechnologists take part as examiners; however, in the debate session and consensus there are 6 other experienced cytotechnologists and 1 pathologist headed by Dr. José Antonio Palet Guzmán, Chief of the Department of Cytopathology. These people complete the diagnosis team at the HRRB.

A patients' treatment depends on if it is possible to decide on the presence of an AGC. For this reason, it is necessary to deeply analyze the cytological findings suggestive of benign changes and call them “negative for high grade epithelial Cancer injury” when possible. However, human eye interpretation is inconclusive in the cytological image analysis due to the different criteria on making a diagnosis of AGC. On the other hand, the different interpretations of a patient's clinical background will result in a consensus, since subjectivity appears on the scene at the moment of making a decision for a positive diagnosis of CN. This expert system allows unified criteria among many examiners as long as enough correct data is provided. This means that all the blanks in the interface must be filled by the user. If any error is given to the expert system, its reliability will decrease considerably.

### 2.2. Risk Factors for CN

The origin of cervical cancer is multifactorial. The most outstanding risk factor is infection of human papillomavirus (HPV). However, the patient's ages, and the early beginning of his sexual relations are factors to be considered extremely important [7].

Multiparity is another risk element to be considered. According to published studies, it has been proven that an immunologic and folic acid depression occurs. The more pregnancy numbers, the higher the risk of intraepithelial neoplasms [8]. In the same way, the age of the first gestation is of vital importance in CN diagnosis, since it helps to estimate the evolution of this disease. Having multiple sexual partners is another important factor, primarily if contraceptive methods are hormonal instead of barrier methods.

Some other important factors are the presence of sexually transmitted diseases (STD) and cervical injuries found in Pap smears (Pap smears is a test in which cells from the uterine cervix and endocervix are sampled, spread on a glass slide, stained, and interpreted by a cytotechnologist or pathologist based on the Bethesda system abnormal values. The PS is the “gold standard” method for early detection of HPV, herpes, trichomonad infections, CIN/dysplasia, and cervical CA accuracy PS interpretation is a subjective art based on experience of the screening cytotechnologist or pathologist. http://medical-dictionary.thefreedictionary.com/Pap+smear).

### 2.3. Detection and Diagnosis

There are cases that even though cytology is positive, a patient does not show malignant or precursor injuries. In other cases, cytology is negative, and in further checking, malignant anatomical changes are found which are not identified at first, incurring in false positives or false negatives [9]. 

The current suggested terminology for reporting results in cervical cytology is the Bethesda system. This system was developed for the National Cancer Institute (NCI) in 1988, with the purpose of providing a uniformed terminology to facilitate communication between the pathologist and gynecologist. The main purpose of this system is to inform the gynecologist the most information available to be used in a patients' treatment by the means of a descriptive report in which all the cytological aspects must be included (hormonal, morphologic, and microbiological levels) [10].

### 2.4. A Literature Review

As a part of this research, many other related works were consulted. These pieces of work are based on different technological and methodological techniques.

Deligdisch et al. present a classification of human papillomavirus (HPV) in teenagers. Here, they identify histological changes in the cervical uterus of infected teenagers with HPV and contrast their results with the results of older patients. The analysis presented was carried out by the means of image treatment techniques, and their final contribution was to assess cytopathic effects of HPV in teenagers [11]. 

Torres et al. assess the role of the main risk factors linked to high-risk squamos intraepithelial injuries in Cauca, Colombia. The methodology used in this research consists of statistic case studies and checkups. In addition, this study confirms the relation between HPV and the risk of cervix neoplasm. Data suggest that multiparity and exposure to carcinogens present in smoke from firewood are risk cofactors when HPV is presented [12]. 

Arcos' research results show that it was possible to classify 75 vaginal epithelial cells after using operations such as preprocessing, segmentation, and representation of digital images. Software was built which allows you to analyze cell classification parameters, such as relationship between nucleus cytoplasma, staining, saturation, luminance, and shape and position of the nucleus, with the purpose of identifying superficial, intermediate, basal, parabasal, and dysplastic cells [13]. 

In her research, Machorro describes the development of an expert system that eliminates subjectivity that may occur in the different viewpoints among colposcopy specialist. After this, the information is presented to gynecologists as an alternative support for making a decision in diagnosing the type of CN precursor injury based on fuzzy logics and colposcopic images processing [14]. 

Zarandi et al. explain a fuzzy expert system's performance for human brain tumors diagnosis by using T1 contrast magnetic resonance imaging. Fuzzy T2 image processing proposal has four different modules: preprocessing, segmentation, feature extraction, and approximate reasoning. The expert system was validated to prove its accuracy in real situations. The results showed that the system is superior in recognizing brain tumors and its grade [15]. 

In 2011, Tan presents a guide to facilitate the implementation of the Image Guided Brachytherapy (IGTB) which is one of the top-end techniques for cervix cancer treatment. This research was originally published by the Royal College of Radiologists (RCR) in 2009 [16]. 

Petric et al. ran a test in 13 patients in which high-risk-CTV (HR-CTV) was outlined by two observers in T and PT MR image plane, respecting the GYN GEC-ESTRO recommendations for 3D-image based cervix cancer brachytherapy. Contouring time was measured. HR-CTV sizes were compared, and conformity index (CI) was assessed. Inter observer variations in contour extent along eight radial directions were compared between delineation planes. After applying a standard treatment plan, an intercomparison between DVH-parameters V100, D90, and D100 for the HR-CTV was carried out [17]. 

Kande et al.'s, paper presents a novel approach for automated segmentation of the vasculature in retinal images. The approach uses the intensity information from red and green channels of the same retinal image to correct nonuniform illumination in color fundus images. Matched filtering is utilized to enhance the contrast of blood vessels against the background. The enhanced blood vessels are then segmented by employing spatially weighted fuzzy c-means clustering based thresholds which can well maintain the spatial structure of the vascular tree segments. The proposed method's performance is evaluated on publicly available DRIVE and STARE databases of manually labeled images. On the DRIVE and STARE databases, it achieves an area under the receiver operating characteristic curve of 0.9518 and 0.9602, respectively, being superior to those presented by state-of-the-art unsupervised approaches and comparable to those obtained with the supervised methods [18].

Shin et al. proposed the Index-Blocked Discrete Cosine Transform Filtering Method (IB-DCTFM) to design an ideal frequency range filter on DCT domain for biomedical signal which is frequently exposed to specific frequency noise such as motion artifacts and 50/60 Hz power line interference. IB-DCTFM removes unwanted frequency range signals on time domain by blocking specific DCT index on DCT domain. In simulation, electrocardiography, electromyography, and photoplethysmography are used as a signal source, and FIR, IIR, and adaptive filters are used for comparison with proposed IB-DCTFM. To evaluate filter performance, we calculated signal-to-noise ratio and correlation coefficient to a clean signal of each signal and filtering method, respectively. As a result of filter simulation, average signal to noise ration and correlation coefficient of IB-DCTFM are improved about 75.8 dB/0.477, and FIR, IIR, and adaptive filtering results are 24.8 dB/0.130, 54.3 dB/0.440, and 29.5 dB/0.200, respectively [19].

Rogel et al. proposed that French uterine cancer recordings in death certificates include 60% of “uterine cancer, Not Otherwise Specified (NOS)”; this hampers the estimation of mortalities from cervix and corpus uteri cancers. The purpose of this work was to study the reliability of uterine cancer recordings in death certificates using a case matching with cancer registries and estimated age-specific proportions of deaths from cervix and corpus uteri cancers among all uterine cancer deaths by a statistical approach that uses incidence and survival data [20].

Thomas proposed that external irradiation and brachytherapy are curable for the treatment of carcinoma of the cervix. The intention of radiotherapy is to optimize the irradiation of the target volume and to reduce the dose to critical organs. The use of imaging (computed tomography and magnetic resonance imaging added to clinical findings and standard guidelines) is studied in the treatment planning of external irradiation and brachytherapy in carcinoma of the cervix. Imaging allows for individualized and conformal treatment planning [9].

Zwahlen et al. purpose to determine the feasibility and benefits of optimized magnetic-resonance-imaging- (MRI-) guided brachytherapy (BT) for cancer of the cervix. A total of 20 patients with International Federation of Gynecology and Obstetrics Stage IB-IV cervical cancer had an MRI-compatible intrauterine BT applicator inserted after external beam radiotherapy. MRI scans were acquired, and the gross tumor volume at diagnosis and at BT, the high-risk (HR) and intermediate-risk clinical target volume (CTV), rectal, sigmoid, and bladder walls were delineated. Pulsed-dose-rate BT was planned and delivered in a conventional manner. Optimized MRI-based plans were developed and compared with the conventional plans. MRI-based BT for cervical cancer has the potential to optimize primary tumor dosimetry and reduce the dose to critical normal tissues, particularly in patients with small tumors [21].

Gordona et al.'s work is focused on the generation and utilization of a reliable ground truth (GT) segmentation for a large medical repository of digital cervicographic images (cervigrams) collected by the National Cancer Institute (NCI). The NCI invited twenty experts to manually segment a set of 939 cervigrams into regions of medical and anatomical interest. Based on this unique data, the objectives of the current work are to (1) automatically generate a multiexpert GT segmentation map; (2) use the GT map to automatically assess the complexity of a given segmentation task; (3) use the GT map to evaluate the performance of an automated segmentation algorithm. The methods and conclusions presented in this work are general and can be applied to different images and segmentation tasks. Here they are applied to the cervigram database including a thorough analysis of the available data [22].

Xue et al. proposed that segmentation is a fundamental component of many medical image-processing applications, and it has long been recognized as a challenging problem. In this paper, they report our research and development efforts on analyzing and extracting clinically meaningful regions from uterine cervix images in a large database created for the study of cervical cancer. In addition to proposing new algorithms, they also focus on developing open source tools which are in synchrony with the research objectives. These efforts have resulted in three Web accessible tools which address three important and interrelated subtopics in medical image segmentation, respectively: the Boundary Marking Tool (BMT), Cervigram Segmentation Tool (CST), and Multi-Observer Segmentation Evaluation System (MOSES) [23].

As a result of the literature review, we can observe that, even if the uses of techniques of expert systems such as fuzzy logics and images interpretation are common in medicine, the study of atypical gland cells (AGC) is hardly explored.

In recent years, FL has been used in different types of machines, systems, and software, among other applications of everyday life. Some examples are lift controls, earthquake engineering, and even video games. However, among other applications that allow this technique, at the same time one of the most innovative and complex, is medicine. On the one hand, it is possible to take advantage of the imprecision tolerance, and on the other hand, to unify vital criteria for the decision making process. The main contribution of fuzzy logic relies on the easiness to deal with complex, hard to define, or lack of math-model problems that would allow for their solution. Research works such as that of Machorro (2010) show us how this technique lets us unify criteria and reduce subjectivity among different points of view of specialists on an issue.

Although image interpretation is considered as an “open field” in research, the progress in this area has not been by itself but has been mostly linked to other areas. The rise of digital image processing (DIP) can be seen in areas such as astrology, geology, electronics, and, most importantly for us: medicine. Image interpretation allows us to obtain data that the human eye is not able to perceive in CAT scans, X-rays, or cytological image, for cervix cancer detection.

Cancer cells research is nothing new, since the techniques mentioned above have made an incursion in the study of this disease since decades ago. For instance, we can talk about researches comprising since HPV as a risk factor in contracting cervix cancer by the means of statistical analysis as described in works of Deligdisch et al. and Torres et al. Some other researchers such as Arcos and Zarandi et al. have applied image interpretation to identify and classify cervical cells and cerebral tumors. On the other hand, works by Thomas, Zwahlen et al., Gordona et al., and Xue et al. focus on segmentation techniques for countless medical applications for image interpretation; for instance, regions of clinical interest extraction, MRIs guided by brachytherapy, or even optimized radiation of CTV (clinical target volume?), and reduction doses, on critic organs, among others. 

However, a problem for experts on the matter is atypical cells analysis. In this case, atypical gland cells (AGC) are presented when diagnosing cervix cancer (consider revision in Spanish). The main contribution of this research is the study of AGC for their consideration within the process of cervix cancer diagnosis, and at the same time, the reduction of false positives and false negatives that come along with these types of diagnosis.

## 3. Development of the Expert System

**Figure 3:**
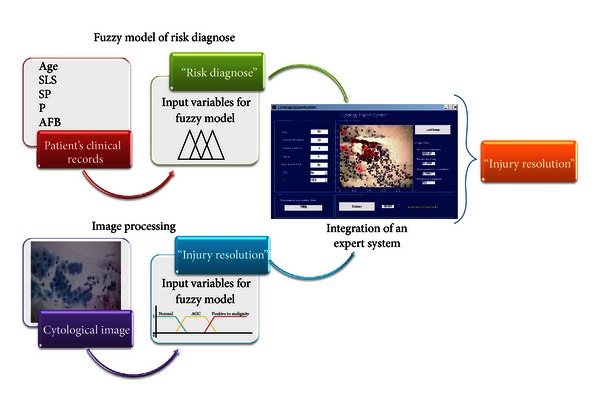
Integration of the expert system.

This expert system was developed in 3 phases or stages (Figure 3). The first stage is the risk diagnosis; this stage consists of a fuzzy model that measures the risk for contracting CN according to the patient's clinical records. The second stage is the interpretation of a cytological image where a process and an image interpretation are carried out in order to obtain common parameters in images with regular diagnosis, cancer, and in squamous cell Atypical Glandular Cells (AGC). The third stage of the expert system links the obtained results in the fuzzy model of the first stage with the results of the images processing in order to create a second fuzzy model, in which injuries can be classified. This classification is given by three output variables: no alterations, atypical glandular cells (AGC), and malign positive.

The expert system uses Matlab's toolbox for the development of the fuzzy logic model and image interpretation. A graphic interface that integrates Matlab's tools (fuzzy logic and image interpretation) was created so that the expert system can be user-friendly.

### 3.1. Fuzzy Model of Risk Diagnose

In the decision-making process for CN diagnosis, risk factors are considered essential when an atypical glandular cell (AGC) occurs, since a diagnosis can encourage a debate among specialists, and this could eventually affect the clinical procedure followed by doctors that could lead to false positives or false negatives. As a result, the expert system starts with a fuzzy logics model that determines if a patient is at risk of developing CN according to their clinical records. 

The fuzzy rules combine one or more fuzzy input sets (background) and establishe association with output fuzzy sets (consequence risk). This rule format is known as the Mamdani (Mamdani FLC (Fuzzy Logic Controler) proposed by Mamdani and Assilian in 1994. This controller uses the error *e*(*k*) and the change of error Δ*e*(*k*) to produce changes in the output function of the controller.) type and works with a fuzzy controller that settles up a system on its work area.

As mentioned earlier, the variables to be used are obtained directly from the patient's clinical records and according to what specialists' consider significant to their diagnosis of CN. Identified variables and their sets are shown in Table 1.

The output variable for this model is considered with only two sets: positive risk and negative risk. These sets are given by independent triangle graphs; this means that a result will be negative if the gap is between 1 and 1.5, or positive if the output variable is superior to or equal to 1.51. 

**Figure 4:**
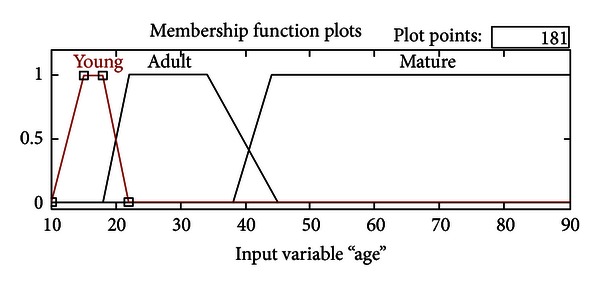
Modeling of variable “Age.”

The modeling of these variables was made with the Matlab software. The intervals used for this model were provided by specialists based on their expertise. One example of this model is represented with the variable labeled “age” (Figure 4):

(1) μyoung(E)={0;t<101−15−t15−10;10≤t≤151;15≤t≤181−t−1822−18;18≤t≤220;22>t,μadult(E)={0;t<181−22−t22−18;18≤t≤221;22≤t≤341−t−3445−34;34≤t≤450;45>t,μmature(E)={0;t<381−t−4040−38;38≤t≤401;40>t.

The creation of the rules of inference in the system was determined based on the linguistic input variables and their fuzzy sets. The combination of the seven variables along with their sets brought about 972 rules. After these inference rules were validated by specialists, only 360 rules were left to run the system. Some of these rules can be seen in Table 2.

The output value according to each reference rule was determined by the specialists.

The defuzzification process that uses the Matlab software calculates the center of gravity of images (centroid method) that are generated at the moment of decomposing each of the linguistic input variables; this means that this process analyzes the existing association between the membership grades of two joint sets and calculates the surface of each resulting image. In other words, the centroid method uses the classic form, in relation to the abscissa axis, the center gravity in rectangles is located in the middle of its base, and for triangles, it is found at the third part of its base according to the opposite side of the angle created by the hypotenuse and the base. 

Finally, the total centroid is calculated. To obtain this variable, the addition of the surface product of each figure is divided by its centroid into the total surface. 

### 3.2. Image Processing

**Figure 5:**
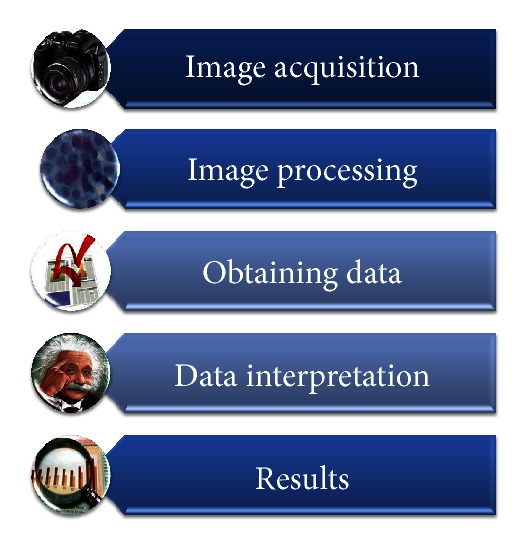
Image processing.

Image processing consists of many different stages that make it possible to identify useful input parameters for an expert system enabling it to determine cancer precursor injuries. To provide an overview about image processing, Figure 5 is shown and explained. 

Images processing consists of the use of techniques on one or more images in order to obtain data or specific features. Applied techniques in this research focus on the identification of endocervix cell nuclei, as well as their size, staining, shape, and interpretation. These features are further used as linguistic or input variables of the fuzzy logics model that make up the expert system of this research.

**Figure 6:**
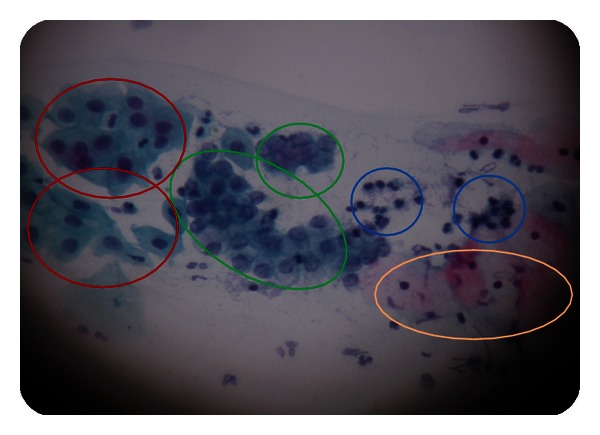
Benign cytological findings.

This study was based on cytological images. Cytological images are classified in three diagnoses: normal or negative to malignant, atypical glandular cells (AGC), and adenocarcinoma. When analyzing an image, specific features are expected to be found. These features or sets of features will define the sort of condition. In Figure 6, you can observe some of the important findings. Outlined in green you will find normal or negative to malign cells. Outlined in red, you can see metaplastic or evolving cells, which can turn into cancer. Outlined in blue, there are the inflammatory cells (also known as mast cells), which are not so relevant for analysis and diagnosis of an injury. Finally, in light orange you can observe exocervical cells that in this research we will not be looking at since these types of cells are studied in histology.

The findings which were discussed earlier are the characteristics that are intended to extract from each image. These features are considered by specialists as fundamental for CN diagnosis. The image-processing techniques applied in the Matlab software for this work will be discussed up next. 

*(a) Level Enhancement and Embossing.* In this first stage of the process, it is intended to improve the color levels of the image by adjusting its brightness and contrast for the purpose of stressing the interest features in it. Applying any of these adjustments, you may observe noticeable changes in the images. In the next diagram, we can see the original image in the left part, just as a specialist would see through the microscope. On the other hand, on the right side we can see the same image with the first level adjustment applied. The image is acquired directly from the microscope through a photographic camera which contains low default levels of color, brightness, and contrast. For this reason, the levels must be adjusted in order to get a better quality of the image and outline the areas of interest. The levels of brightness and contrast were adjusted according to the specialist's experience and appreciation. Once the camera was ready according to the specialists' requests, the camera adjustments were set for the rest of the photographs. 

**Figure 7:**
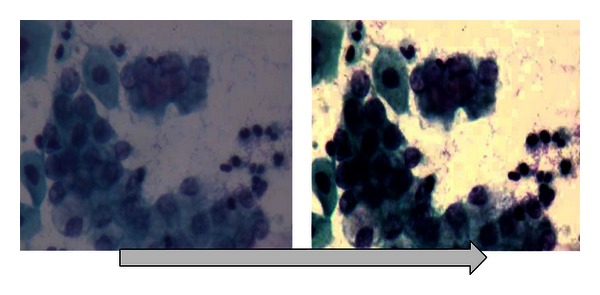
Level enhancement and embossing.

In Figure 7, you can immediately identify the darkening of the nucleus and the main interest feature, as well as the cytoplasm definition. The background of the image could be mistaken with the cytoplasm when adjusting brightness and contrast levels, since this adjustment makes it clearer to identify. Moreover, the edge of the cell membrane is better defined for further analysis. 

**Figure 8:**
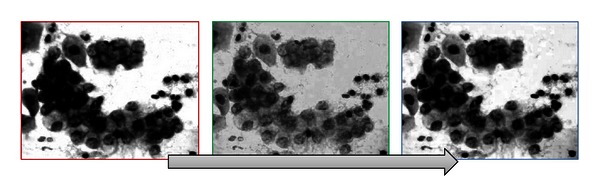
Color separation on RGB.

*(b) Color Separation.* Once the image has been adjusted, it is divided into three channels, or its color composition, this means, red, green, and blue channel, best known as RGB. In Figure 8, you can observe the channels composing the image. 

The following step in the image processing is where nuclei are going to be identified. This step was decided to be carried out in the green channel by the specialists in the HRRB. The specialists based this decision on their expertise since this channel keeps and better marks the interest features. 

**Figure 9:**
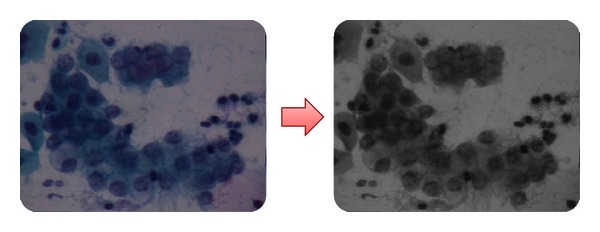
Image converted into grayscale.

The grayscale technique will be used to obtain some other parameters such as nuclei staining, dimension, and nucleus shape. In Figure 9, you can see the image converted into grayscale.

**Figure 10:**
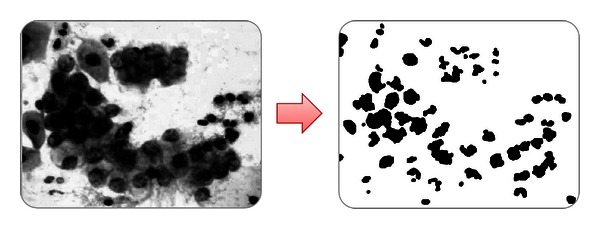
Threshold implementation for nuclei identification.

*(c) Image Threshold.* A threshold is a filter that works by dividing the image into two colors (black and white) and uses a middle spot or interest spot that can define a separation threshold in both colors according to the luminosity of the original image. If this is the case, threshold is used to divide the nucleus in an image, in order to obtain definite nuclei. In Figure 10, you will observe an image with a threshold over a green channel.

**Figure 11:**
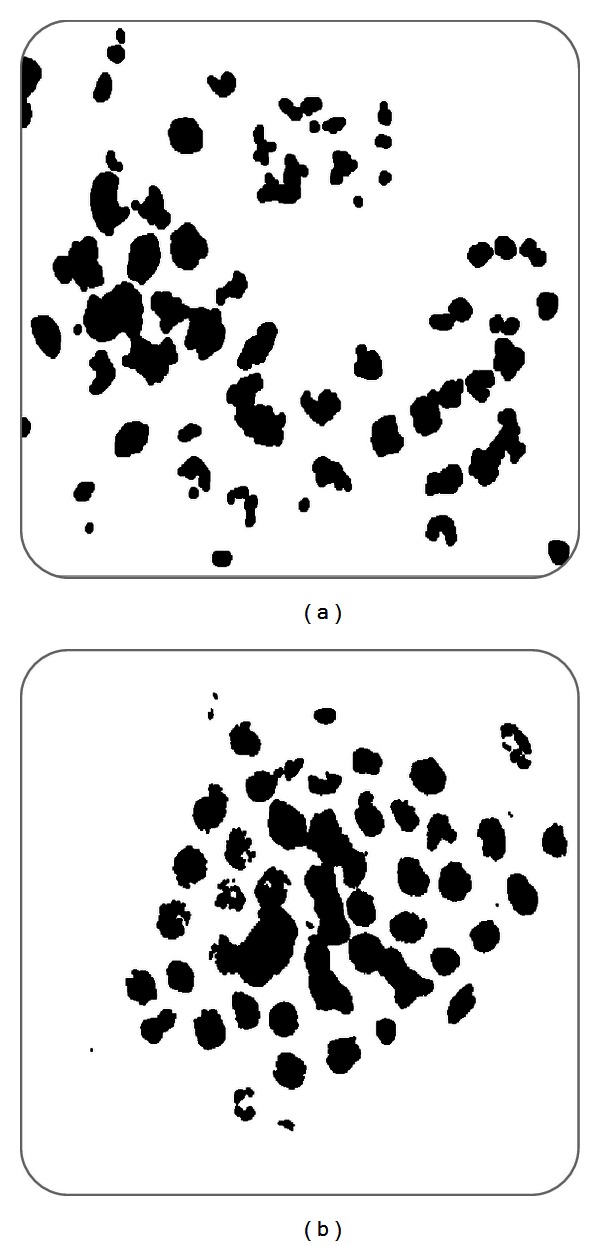
Identification of nuclei.

*(d) Morphologic Operations.* In image processing, morphology is a set of techniques based on shapes. This technique is applied to an input image (Figure 11) but does not affect its output. The set of techniques are dilation, erosion, closure, and disconnection.

**Figure 12:**
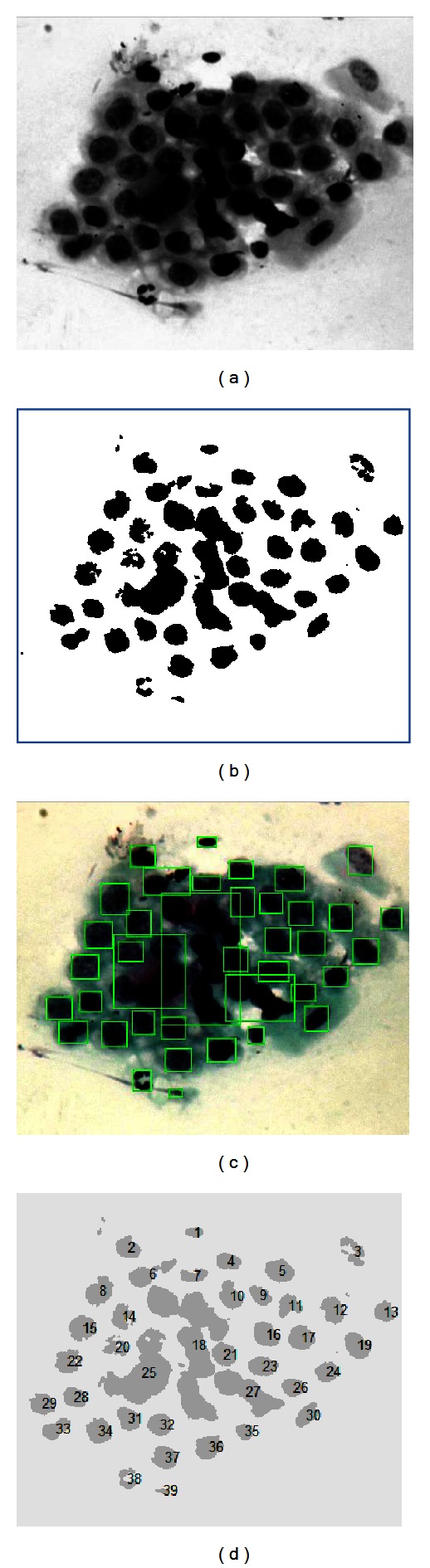
Identification and labeling of nuclei.

*(e) Labeling.* This part of the image processing consists in identifying the number of objects that can be found in an image. Later, it is analyzed in order to obtain the area, perimeter, and centroid of the identified nuclei. This stage is performed in the green channel by using threshold and morphologic operations in order to determine the nucleus edges, which later will be used as a study object for parameter determination. In Figure 12, the process to identifying image nuclei can be observed. 

**Figure 13:**
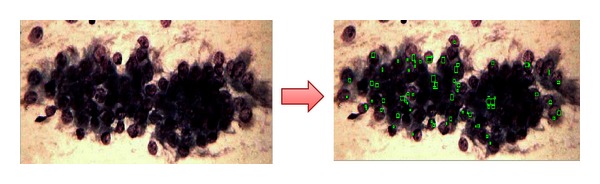
Identification of nucleolus.

By using a similar process to the prior, the number of nucleolus is obtained (Figure 13).

In order to deeply know the content of an image it is necessary to perform an analysis of the data gathered during its process. Furthermore, during this process it is possible to request data such as pixel size, nuclei diameters, symmetry, or eccentricity where specialists can observe features of interest.

One form of data interpretation in a clearer way is by using descriptive statistics. With this discipline we can obtain parameters that describe image information. For each data, images tend to settle in different ways; however, the images with the same condition tend to be alike. For this reason, after all the images from each condition are analyzed, the following variables are determined.

(i) *Nucleus dimension:* this refers to the size of each of the identified nucleus. To determine this parameter, the area of nucleus column is used and then you calculate the average area of the nucleus that can be seen in the image:
  (2) $$\begin{array}{c} DN=\frac{\sum \text{nucleus}\;\text{dimension}\left(\text{pixels}\right)}{\text{number}\;\text{of}\;\text{nucleus}\;\text{detected}}. \end{array}$$
(ii) *Nuclei staining:* this refers to the pigmentation of the detected nuclei, that is to say how darkened the nuclei are, because the darker they are the more hyperchromatic they are considered. In order to obtain the nuclei staining appearing in an image, the nuclei pixel size column is used, and then the general average of the nuclei is calculated:
  (3) $$\begin{array}{c} TN=\frac{\sum \text{pixel}\;\text{average}\;\text{of}\;\text{each}\;\text{nucleus}}{\text{number}\;\text{of}\;\text{nucleus}\;\text{detected}}. \end{array}$$
(iii) *Homogeneous nucleation:* it is considered as homogeneous when identified nuclei are alike among them, meaning, when they are regular. The nuclei are considered to be regular when they tend to be rounded. In order to calculate homogeneous nucleation, the eccentricity column is used, and this column suggests that if eccentricity value in the nuclei tends to be 0 it is more rounded, but if the value tends to be 1 it is more elongated.
(iv) *Nucleolus presence:* one important part of the diagnostic criteria in CN is the ability to identify nucleolus in an image. Along with the image processing, a numeric data of the amount of identified nucleolus is obtained.

### 3.3. Integration of an Expert System: Fuzzy Model “Injury Resolution” 

 An expert system is that which is capable of emulating a decision-making process involving a human being with inaccurate information. Also, it is capable of making a decision with incomplete information just the way a specialist in a subject would. The expert systems based on fuzzy logics are able to transform linguistic variables into numeric values by using fuzzy sets, in which each variable belongs in a certain degree to another Fuzzy set in a different degree. The basis of this knowledge is built on a specialist expertise through rules represented with phrases such as: if… then; if not… then, known as Inference Engine. In a similar way, just as the fuzzy model “Risk,” the fuzzy model “Injury Resolution” was carried out within the Mamdani type. 

**Figure 14:**
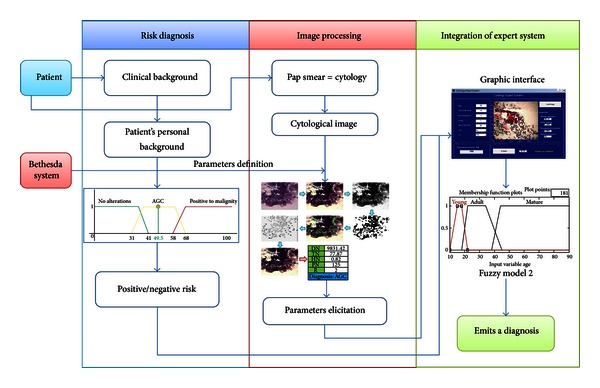
Expert Systems Process.

The integration of the expert system during this research was carried out through a graphic interface in which the user only needs to type a patient's clinical background data such as age, beginning of sexual life, number of children, and so forth. Then, upload the cytological image sample to be analyzed and finally press the bottom “Evaluate.” The output variable (risk) and the result from the image process constitute the input variables of the second fuzzy model, which at the same time brings out the numeric and linguistic value that can suggest a diagnosis. The integration process can be observed in Figure 14.

The resulting data from the latter processing will provide parameters that will be turned into linguistic variables or input variables for the fuzzy model. The input variables to the fuzzy logics system are in Table 3.

**Figure 15:**
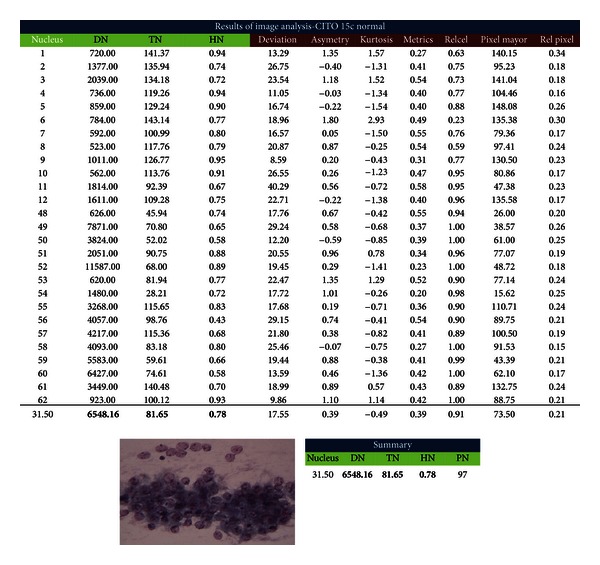
Image processing results.

As mentioned earlier, through the image processing it is possible to request data such as pixel size, nuclei diameters, symmetry, or eccentricity. Each of these data provides interest information to specialists since the analysis is made nucleus per nucleus (Figure 15).

One way to obtain parameters that describe an image's information is by Interval Estimation. Among images with the same condition, the data averages happen to be very similar. Due to the information provided by the image processing, theses intervals for the first four variables were set by using confidence intervals (Table 6). This means that the confidence interval of the average of each set would represent 100% of the set's membership in relation to the variable.

**Figure 16:**
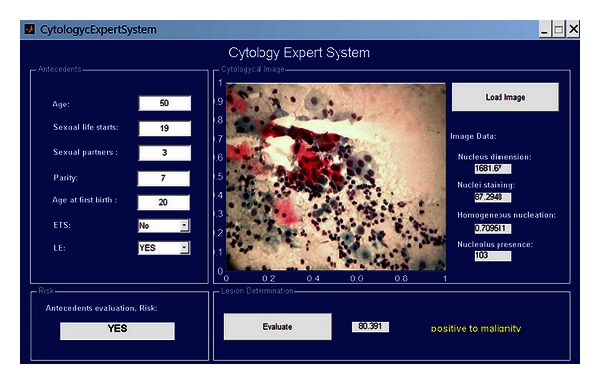
Expert system interface.

Each set for defuzzification of a variable was modeled in relation to the confidence intervals. Up next, the variable modeling “Nuclei Density” is presented (Figure 16), (4):

(4) μsmall(DN)={1;0≤t≤50001−t−50006000−5000;5000≤t≤60000;t≥600,μmedium(DN)={0;t<45001−6800−t6800−4500;4500≤t≤68001;6800≤t≤90001−t−900010000−9000;9000≤t≤100000;t≥10000,μlarge(DN)={0;t≤90001−9900−t9900−9000;9000≤t≤99001;9900≤t≥25000.

One hundred and eight inference rules were created (5) which were validated by specialist doctors with the purpose of determining that all of them were feasible in relation to the variables' combination; for example, if in an image a “dark” nucleus is found it is not possible to detect the nucleolus; therefore, this rule turns out invalid:

(5) DM(3)×TN(2)×HN(2)×PN(3)×R(2)  =108  Inference  Rules.

Some of these rules can be seen in Table 4.

## 4. Test and Results

A graphic interface allows interaction with users in an easier way within the system. The expert system interface presented here (Figure 16) includes a background section (upper left), in which numeric and linguistic data is typed. Then at the upper right, an “upload image” button is included; here the image is selected from stored images from which study cases are generated. The background evaluation box (lower left) allows one to know if the patient has or does not have risk of contracting CN according to the clinical analysis. This information can be consulted by pressing the “evaluate” button in the “Injury Resolution” section. It is in this section that the numeric and linguistic complete analysis value of a case will appear (background + cytological image), and in the same way, numeric values for each parameter of the image processing (image data) will be shown. 

The numeric value of the image processing is proportional to the malignancy grade of each case; however, the injury grade of each sample such as cervical intraepitelial neoplasm (CIN) I, II, and III was defined by specialists. This means that the system is capable of offering a diagnosis to cases with no variation (NV), atypical glandular cells (AGC), and positives to malignity, which are considered as the basis for CN diagnosis. These positive cases can be classified as infiltrating, invasive, adenocarcinoma, and so forth according to time and gravity of the injury. If an injury reoccurs or has not been treated before, specialists' expertise intervene. 

The system tested the cases received at the hospital from 2010 to 2012. Given the fact that AGC are not common, these cases were recorded by the hospital and labeled as “suspicious” of AGC. It is important to mention that only a few patients present cases where AGC are involved. 

In the first model validation of the fuzzy system (risk diagnosis), 20 gynecologic cases of patients from Rio Blanco's Regional Hospital (HRRB in Spanish) were considered, since these cases already had a diagnosis. These cases were the population sampling. The criteria for choosing these cases was that they had a cytological diagnosis, which was no longer than 1 year, and at the same time, their lamella was well preserved for a later photograph. 

These cases were selected by the specialists because in 2011 a new microscope with photograph camera was acquired by the hospital, and therefore, the most recent lamella and complete data record cases were chosen.

Note: not all of the cases were positive to AGC, since the first diagnosis was just “suspicious for AGC.” The latter was clarified further in the decision-making process named “session” where some cases where dropped as AGC. During the running test of the expert system, the final diagnosis emitted in the session at the HRRB was taken into consideration. 

*The system hypothesis is as follows.*

*According to the clinical backgrounds of a patient, if she is at risk of developing Cervical Cancer, the result of the cytology diagnose must be positive, whereas, if the result of the system shows that there is no risk in developing Cervical Cancer, the diagnosis of the patient must be negative to cancer in relation to its last cytology.*

As a result at the end of the validation of the system, we had a 100% correlation between the fuzzy model “Risk” and the cytology diagnosis emitted by the specialists. The system is valid to determine if a patient has a risk of developing CN only by looking into her clinical background.

The final system was tested with 10 diagnosed cases because they were the only ones that had a complete file at the moment of the test run. Another criterion was that since these cases were atypical there were not many of these kinds available. These 10 cases selected by the Head of Pathology were cases that were not able to be diagnosed at first and it was necessary to examine them again. Once the second tests were made and diagnoses were emitted, these cases became immediate candidates to run the expert system. The period in which these cases were taken for studying and running of this research was from May 2010 to January 2012. 

The presence of risk for these cases was assessed with the first fuzzy model “Risk,” in such a way that, in order to evaluate the model “Injury Resolution”, a different output value was used in relation to the first model. 

Up next, 3 out of the 10 cases tested are presented by the expert system. All of the cases were treated as AGC when examined for the first time by the specialists in the cytology sample, whereas their clinical backgrounds did not show any risk of contracting CN. 

### 4.1. Case  1: Normal Cytology, Negative to Malignity

**Figure 17:**
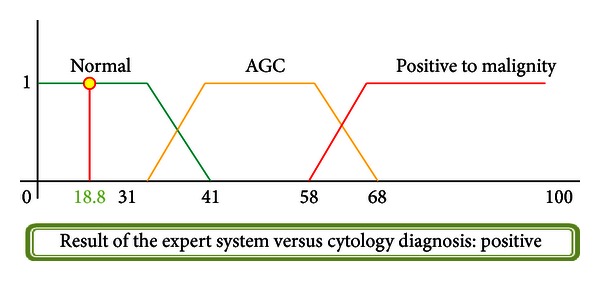
Injury resolution.

A 47-year-old woman, beginning her first sexual relation at the age of 23, 2 sexual partners and 1 gestation completed at the age of 27. No previous sexual transmission disease (STD) recorded. No cervical injuries found during exploration. Cytology diagnosis provided by the specialist: Negative to malignity = No alterations. The result of the system is shown in Figure 17.

### 4.2. Case  5: Cytology, Positive to Malignity, AGC

**Figure 18:**
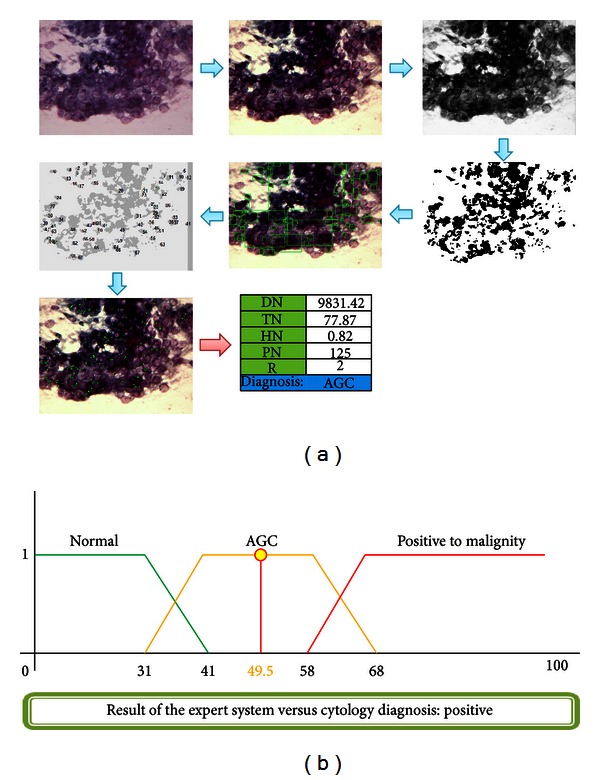
Injury resolution.

A 43-year-old woman, beginning her first sexual relation at the age of 16, 1 sexual partner and 7 gestations completed at the age of 28. No previous sexual transmission disease (STD) recorded. No cervical injuries found during exploration. Cytology diagnosis provided by the specialist: AGC. The result of the system is shown in Figure 18.

### 4.3. Case  8: Positive Cytology, Adenocarcinoma

**Figure 19:**
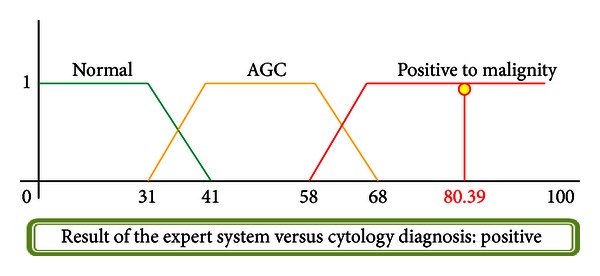
Injury resolution.

A 50-year-old woman with first sexual relation at the age of 19, 3 sexual partners, and 7 gestations completing the first at the age of 20 via vaginal channel. No STD recorded. Cervical injuries found during exploration. Cytology diagnosis provided by the specialist: Adenocarcinoma = precursor injury of CN. The result of the system is shown in Figure 19.

In Table 5, the obtained results from the 10 diagnosed cases in HRRB are shown. Although it is true that the results obtained from the tests cannot show a significant reduction of false positives or false negatives, in these cases, 100% correlation between the doctors and the system was reached; however, a further test with more cases is expected. It is expected that with the implementation of this tool the percentage in the reduction of false positives and false negatives can be quantified.

The expert system is a supportive tool in the cervical cancer diagnoses at the HRRB, but the final decision is made by the specialist. However, in this section it is proven that tools and techniques such as image processing and fuzzy logics are as capable of emulating a decision-making process as much as a specialist. The image processing turns out to be a very interesting tool since it is through these kinds of techniques that detailed and accurate information can be consulted rather than just the subjectivity of the human eye in manual diagnosing. At the same time that fuzzy logics is a helpful tool for meeting criteria, it also helps model knowledge and expertise from specialists. Finally, the implementation of interfaces helps users to understand and interact in a simpler and easier way with the expert system.

As far as the data is reliable, the system will be able to make a diagnostic suggestion which helps the specialist to offer patients an opportune and appropriate treatment.

## 5. Conclusions

Cervix neoplasia (CN) is a public health matter; it is one of the most important causes of death from neoplasia among female population. Nevertheless, this type of cancer is preventable and its treatment can be considered relatively easy, when the diagnosis is opportune. 

The Pap smear is not a diagnostic test, it is just a screening checkup that differentiates from those women who could have cervical injuries from those who cannot. The results from these tests are not always “accurate.” In many occasions, cytology can be positive, but at the end, the patient may not have cancer precursor or malignant injuries; whereas in other occasions, even though cytology is negative there may be malignant changes that were not first detected. Unfortunately, although this situation is very serious, it is also very common due to the presence of atypical cases in which samples with insufficient features to be diagnosed with CN tend to malignity and cannot be diagnosed as negative to malignancy (atypical gland cells) either. Human eye perception turns out insufficient for the decision making in the diagnosis. This is why a tool that helps doctors perform a more accurate test is of vital importance. Expert systems can be very useful in the decision making for atypical cases. 

An expert system based on image processing and fuzzy logics applied on this type of illness allows a less subjective decision-making process, meeting existing diagnostic criteria in order to have more accurate information of the studied image. The implementation of the two techniques in this work that integrate the expert system are not feasible in isolation, since the parameters obtained through the image processing are not enough to emit a diagnosis. These results may vary from one to another; this means that it is fuzzy logics that integrates these techniques just the way a specialist would. 

The expert system developed in this research has shown a great effectiveness in predicting if a patient could tend to CN according to her background or risk factors. However, there are many incomplete files that affect the efficiency of the system, since it depends on the input data. Knowledge integration from different fields is indispensable for the development of systems capable to emulate the decision-making process of an expert, since this innovates and enriches expert systems.

In the image processing there is a need to take in some considerations at the moment of taking photographs; this means, if the sample o lamella which is going to be studied is polluted, the photograph's quality will be doubtful, and emitted results from the processing may turn out nonvalid or uncertain. The integration of a first model and the image processing as information sources result in a more efficient expert system. Despite the fact that the system shows 100% positive correlation in relation to the specialist, it is necessary to carry out more tests and determine the error percentage that this might have.

In conclusion, the work presented here is considered as a support tool for specialists and is not intended to replace their invaluable labor. Criteria and considerations for each case can be known only by specialists and only they can make decisions based on their expertise. On the other hand, the implementation of an expert system that only requires a computer and software of commercial use makes it a very feasible tool for areas of low resources or marginalized zones. The implementation of the expert system is easy handling for doctors, since the graphic interface turns out to be user-friendly. The system can also be used as a training tool for new doctors, since within it, information, knowledge, and expertise from certified specialists are stored. Finally, it is worth mentioning that the contribution of this research relies on the support in the decision-making process of CN diagnosis. With this, it is expected to reduce the number of false positives and false negatives in cases where atypical cases occur.

## Figures and Tables

**Table 1:** Input variables of fuzzy model of risk diagnose.

Input variables	Label	Geometric shape	Interval
	Young	Trapezoid	(10 15 18 22)
E (age)	Adult	Trapezoid	(18 22 34 45)
	Mature	Trapezoid	(38 44 90 90)

IVSA (onset of sexual activity)	Young	Trapezoid	(10 15 18 22)
	Adult	Trapezoid	(18 22 34 45)
	Mature	Trapezoid	(38 44 90 90)

	Few	Trapezoid	(0 2 3 4)
PS (sexual couples)	Many	Trapezoid	(3 5 9 10)
	Unusual	Trapezoid	(9 15 20 20)

NG (number of pregnancies)	Few Many	Triangular Triangular Trapezoid	(0 0 0.5) (0.51 4 8) (7 8 18 18)

	Young	Trapezoid	(10 15 18 22)
PG (first pregnancy age)	Adult	Trapezoid	(18 22 34 45)
	Mature	Trapezoid	(38 44 90 90)

ETS (sexually transmitted diseases)	No	Triangular	(0 0 0.5)
	Yes	Triangular	(0.51 1 1.5)

LE (cervical lesions)	No	Triangular	(0 0 0.5)
	Yes	Triangular	(0.51 1 1.5)

**R (risk)**	**Negative**	**Triangular**	**(0 0 0.5)**
	**Positive**	**Triangular**	**(0.51 1 1.5)**

**Table 2:** Example of some inference rules.

Nucleus	E	IVSA	PS	NG	EPG	ETS	LE	Risk
1	Young	Young	Little	Null	Young	NO	No	Negative
2	Young	Young	Little	Null	Young	NO	Yes	Negative
3	Young	Young	Little	Null	Young	Yes	No	Negative
4	Young	Young	Little	Null	Young	Yes	Yes	Negative
5	Young	Young	Many	Null	Young	No	No	Positive
6	Young	Young	Many	Null	Young	No	Yes	Positive
7	Young	Young	Many	Null	Young	Yes	No	Positive
8	Young	Young	Many	Null	Young	Yes	Yes	Positive
9	Young	Young	Not common	Null	Young	No	No	Positive
10	Young	Young	Not common	Null	Young	No	Yes	Positive
11	Young	Young	Not common	Null	Young	Yes	No	Positive
12	Young	Young	Not common	Null	Young	Yes	Yes	Positive
13	Young	Young	Little	Little	Adult	No	No	Negative
14	Young	Young	Little	Little	Adult	No	Yes	Negative
15	Young	Young	Little	Little	Adult	Yes	No	Negative
16	Young	Young	Little	Little	Adult	Yes	Yes	Negative
17	Young	Young	Many	Little	Adult	No	No	Positive
18	Young	Young	Many	Little	Adult	No	Yes	Positive
19	Young	Young	Many	Little	Adult	Yes	No	Positive
20	Young	Young	Many	Little	Adult	Yes	Yes	Positive

**Table 3:** Input variables “Inyury resolution (DL).”^1^

Input variables	Label	Geometric shape	Interval
	Small	Trapezoid	(0 0 5000 6000)
Nucleus dimensión (DN)	Medium	Trapezoid	(4500 6800 9000 10000)
	Large	Trapezoid	(9000 9900 25000 25000)

	Clear	Trapezoid	(50 50 80 82)
Nuclei staining (TN)	Media	Trapezoid	(79 82 87 92)
	Dark	Trapezoid	(87 92 150 150)

Homogeneous nucleation (HN)	Regular	Trapezoid	(0.5 0.5 0.74 0.77)
	Irregular	Trapezoid	(0.75 0.8 1 1)

	Null	Triangular	(0 0 2)
Nucleolus presence (PN)	Few	Trapezoid	(2 9 20 30)
	Many	Trapezoid	(24 40 150 150)

Risk (R)	Negative	Triangular	(0.5 1 1.5)
	Positive	Triangular	(1.51 2 2.5)

	Normal	Trapezoid	(0 0 31 41)
(DL) Injury resolution	AGC	Trapezoid	(31 41 58 68)
	Positive to malignity	Trapezoid	(58 68 100 100)

^1^The initials presented in each one of input and output variables come from the translation from Spanish since users are native Spanish speakers. The interface of the expert system is, for now, only available in Spanish.

**Table 4:** Some examples of inference rules.

Rule number	Nucleus dimension	Nucleus tintion	Nucleus homogeneity	Micronucleus presence	Risk	Lesion
1	Small	Light	Regular	Null	Negative	Normal
2	Small	Medium	Regular	Null	Negative	Normal
3	Small	Dark	Regular	Null	Negative	Normal
4	Small	Light	Regular	Few	Negative	Normal
5	Small	Medium	Regular	Few	Negative	Normal

**Table 5:** Final results of expert system.

Final results			
Case	Cytology diagnosis	Result of expert system	Correlation
1	Normal	Normal	Positive
2	Normal	Normal	Positive
3	Normal	Normal	Positive
4	Normal	Normal	Positive
5	AGC	AGC	Positive
6	Adenocarcinoma	Adenocarcinoma	Positive
7	AGC	AGC	Positive
8	Adenocarcinoma	Adenocarcinoma	Positive
9	Normal	Normal	Positive
10	Adenocarcinoma	Adenocarcinoma	Positive

**Table 6:** Confidence interval DN.

	Small	Medium	Large
Data			
Sample size:	5	5	8
Sample average:	3,536.71	7,658.24	13,747.13
Population variance:	1,611,045.74	2,283,910.70	17,225,203.65
Confidence level:	0.99	0.99	0.99
Results			
Alfa (*α*):	0.01	0.01	0.01
*Z*_α/2_ =	2.58	2.58	2.58
*Z*_α_ =	2.33	2.33	2.33

	*μ* interval, *σ* known	*μ* interval, *σ* known	*μ* interval, *σ* known

	2,074.58 **<** *μ* **<** 4,998.84	5,917.35 **<** *μ* **<** 9,399.13	9,967.45 **<** *μ* **<** 17,526.80
	1,402.24 4,658.66	6,045.07 9,831.42	10,080.30 22,828.24
